# Admission neutrophil-to-lymphocyte ratio to predict 30-day mortality in severe spontaneous basal ganglia hemorrhage

**DOI:** 10.3389/fneur.2022.1062692

**Published:** 2023-01-10

**Authors:** Jia Shi, Yu Liu, Li Wei, Wei Guan, Weimin Xia

**Affiliations:** ^1^Department of Neurosurgery, The Third Affiliated Hospital of Soochow University, Changzhou, China; ^2^Department of Rehabilitation Medicine, The Third Affiliated Hospital of Soochow University, Changzhou, China; ^3^Department of Blood Transfusion, The Third Affiliated Hospital of Soochow University, Changzhou, China

**Keywords:** intracerebral hemorrhage, basal ganglia, mortality, NLR, neutrophil, lymphocyte

## Abstract

**Background:**

Spontaneous intracerebral hemorrhage (ICH) usually occurs in the basal ganglia and is highly lethal and disabling. The aim of this study was to evaluate the predictors of 30-day mortality in patients with severe spontaneous basal ganglia hemorrhage.

**Methods:**

This retrospective study included patients with severe basal ganglia intracerebral hemorrhage treated in the Third Affiliated Hospital of Soochow University from 2012 to 2018. Demographic, clinical, laboratory and neuroradiological data were collected. The short-term prognosis was evaluated and divided into death within 30-days and survival over 30-days. We studied the factors affecting the prognosis of patients with severe intracerebral hemorrhage, analyzed the parameters related to neutrophil-to-lymphocyte (NLR) at admission, and evaluated the predictive effect of NLR on 30-day mortality.

**Results:**

A total of 105 patients was included in this retrospective study. The 30-day death group had a larger hematoma, a higher probability of ventricular hemorrhage, a higher ICH score and a lower Glasgow Coma Scale (GCS) score on admission. Meanwhile, the patients in the death group had higher White blood cells (WBC) counts, neutrophil counts, NLRs and C-reactive protein (CRP) levels. The risk factors for 30-day death were related to the ICH volume, GCS score, ICH score, WBC count, neutrophil count, NLR and CRP. The univariate receiver operating characteristic (ROC) curve of the risk factors showed that the NLR had the best prediction performance. Mathematical predictive models for ICH patients showed that the model with NLR had better prediction accuracy.

**Conclusions:**

The NLR is expected to be a potential biomarker for predicting the prognosis of patients with severe basal ganglia hemorrhage.

## Introduction

Stroke is the second leading cause of disability and death in the world (1). As a developing country, the incidence and burden of stroke are also increasing rapidly, and the death related to stroke has become the leading cause of death among Chinese residents (2). In China, the prevalence of stroke increased by 13.2% from 2013 to 2019, with an annual growth rate of 2.2%. The incidence of stroke in adults over 40 years old is 2.58% (about 17.5 million people). The age group with the highest weighted incidence rate of stroke is 70–79 years old, which is 18 folds that of 40–49 years old (3). Spontaneous intracerebral hemorrhage (ICH) is the most serious acute cerebrovascular disease and accounts for 20% of all strokes (4). Only 20% of patients can recover to independent functioning within 6 months after onset, and the mortality rate is close to 60% within 1 year (5). The basal ganglia are located deep in the white matter of the brain and represent an important nerve functional area that is closely related to sensory, motor, visual, behavioral and other functions (6). It also has a high incidence of spontaneous cerebral hemorrhage. Because of the rapid disease development, high mortality and disability rate among patients with severe basal ganglia cerebral hemorrhage (Glasgow Coma Scale, GCS score ≤ 8 and hematoma volume ≥ 30 cm^3^) (7), reliable prediction indicators are needed to help us judge the condition and generate prognoses.

The ratio of the neutrophil count and lymphocyte count in the peripheral blood is known as the NLR, and it can dynamically monitor the body's immune ability and systemic inflammatory state (8). Its ability to predict the clinical outcome has been verified in clinical models of ischemic stroke (9), aneurysm (10), Parkinson's disease (11), and glioma (12). Using ICH models, previous reports found that the NLR of patients was negatively correlated with clinical prognosis (13–15). At present, studies about predicting the prognosis of patients with ICH by NLR mainly focused on patients with mild hematoma volume (16, 17), while few reports had focused on NLR-based prognoses for patients with severe ICH (GCS score ≤ 8 and hematoma volume ≥ 30 cm^3^). Furthermore, the existing literatures roughly included patients with ICH into the study, and did not distinguish the location. It is well known that the location and volume of bleeding are important factors affecting the prognosis of patients.

In the current study, we limited the bleeding location, hematoma volume, and admission status of patients, and only included patients with severe cerebral hemorrhage located in the basal ganglia (GCS score ≤ 8 and hematoma volume ≥ 30 cm^3^), in order to investigate the predictive role of NLR in this highly fatal and disabling stroke.

## Methods

### Study selection

**Figure 1:**
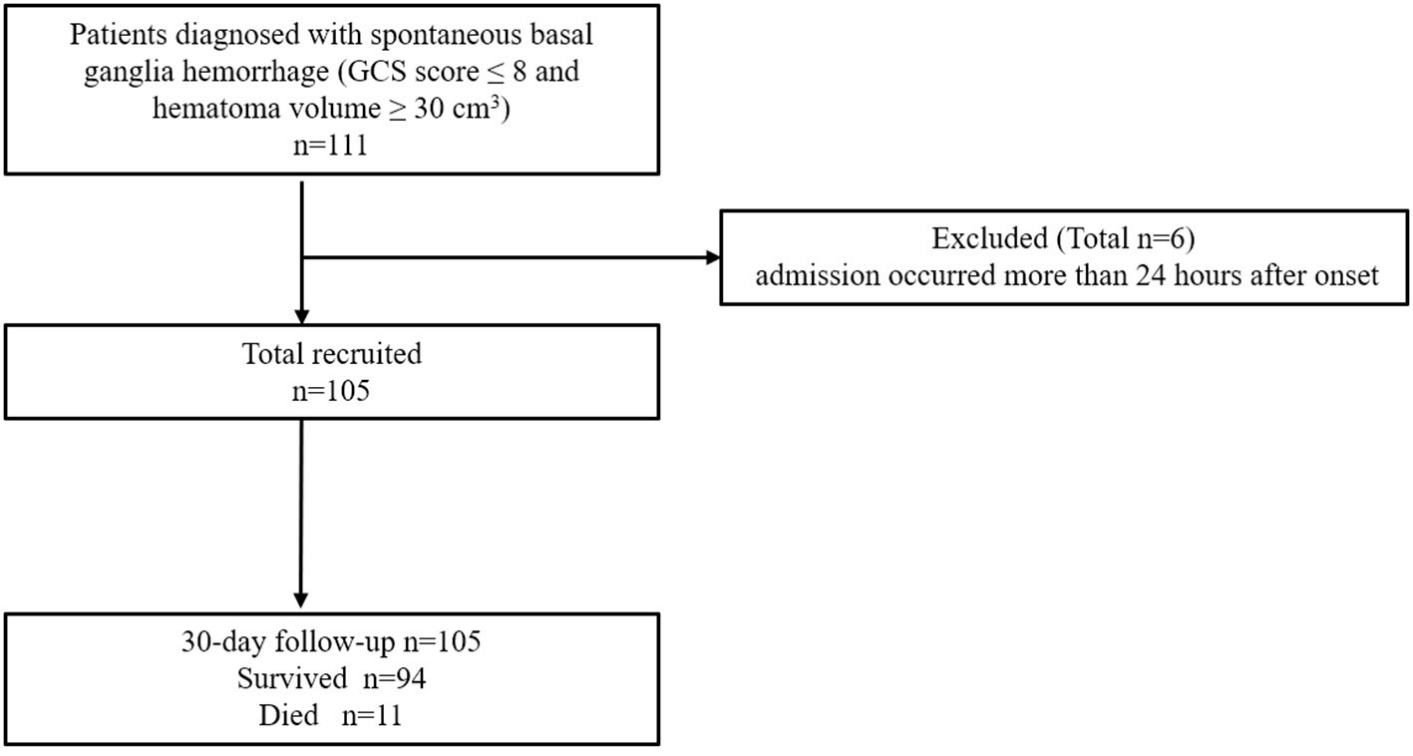
Patient selection flow chart. GCS, Glasgow coma scale.

We retrospectively analyzed 105 patients with severe basal ganglia intracerebral hemorrhage admitted to the Department of Neurosurgery, the Third Affiliated Hospital of Soochow University, from 2012 to 2018 (Figure 1). The inclusion criteria were as follows: (A) head CT images met the diagnostic criteria of cerebral hemorrhage; (B) the hematoma was located in the unilateral basal ganglia; (C) the volume of hematoma, which was calculated according to a previous report (18), was more than 30 ml; (D) admission GCS score was ≤ 8; and (E) age was ≥18 years old. The exclusion criteria were as follows: (A) admission occurred more than 24 h after onset; (B) secondary cerebral hemorrhage in the basal ganglia was caused by cerebral aneurysm, vascular malformation, tumor, trauma, and coagulation dysfunction; (C) anticoagulants and immunosuppressants were used; (D) a history of infection in the past 2 weeks; (E) a history of stroke in the past 6 months; and (F) a history of hematological or malignant tumor. This study was approved by the institutional ethics committee (2021–03). All subjects in this retrospective study were anonymous, and the authors were unable to obtain information that could identify individual participants during or after data collection.

### Data collection

Demographic information, medical histories, GCS and head CT scan results were retrieved from the EMR system. The ICH score was calculated according to the clinical data and CT results (19). All patients provided blood samples on admission and received medical management according to the guidelines of the American Heart Association/American Stroke Association Stroke Council. For comparison, patients were divided into those who survived more than 30-days (*n* = 94) or up to 30-days (*n* = 11).

### Statistical analysis

SPSS software version 25.0 was used for the data analysis. Continuous normally distributed variables are presented as the mean ± SD and were analyzed with independent sample *t*-tests. Categorical variables were expressed as percentages, and the chi-square test was used. To compare the diagnostic efficiency of different parameters, the receiver operating characteristic curve (ROC) and area under the curve (AUC) were analyzed. The diagnostic sensitivity, specificity and accuracy of each variable were calculated, and the optimal cutoff value was determined by the Youden index. Univariate and multivariate logistic regression analyses were performed to detect the correlation between the two variables, and odds ratios (ORs) were calculated to establish the logistic model. ROC and Youden index analyses were performed to establish the best mathematical model for the prediction of ICH patients. The ROCs of all parameters and models were compared by EmpowerStats software. All statistics were tested by a two-sided test, and *p* < 0.05 was considered statistically significant.

## Results

A total of 105 patients were selected from 2012 to 2018, of whom 11 (10.5%) died within 30-days after onset. The patients were classified into different groups according to whether they died within 30-days after onset. The demographic data, clinical characteristics and laboratory parameters of the two groups at admission are shown in Table 1. According to the analysis of the clinical characteristics of the subgroups, the death group had a larger amount of hematoma (median 75, *p* = 0.006), a higher probability of ventricular hemorrhage (72.7%, *p* = 0.049), a higher ICH score on admission (3.0 ± 1.0, *p* = 0.019), and a lower GCS score (median 5, *p* = 0.003) (Table 1). Meanwhile, laboratory examinations found that the patients in the death group had a higher WBC count (17.4 ± 9.0, *p* = 0.002), neutrophil count (14.2 ± 8.2, *p* = 0.004), NLR (median 8.8, *p* = 0.002) and C-reactive protein level (median 49, *p* = 0.012) in their peripheral blood samples, and the differences were significant (Table 1).

**Table 1:** Baseline characteristics of patients according to 30-day outcome.

	**Survived (*n* = 94)**	**Died** **(*n* = 11)**	***p*-value**
**Demographics**		
Male, *n* (%)	61 (64.9)	8 (72.7)	0.605
Age (years)	52.4 ± 14.1	54.5 ± 12.4	0.640
**Medical history**, ***n*** **(%)**		
Prior stroke	11 (11.7)	0 (0.0)	0.230
Hypertension	57 (60.6)	9 (81.8)	0.169
Diabetes mellitus	10 (10.6)	1 (9.1)	0.874
**Admission assessment**		
Duration from onset to hospitalization (h)	3 (2–4)	3 (2–4)	0.484
Right basal ganglia hemorrhage, *n* (%)	37 (39.4)	2 (18.2)	0.169
ICH volume (mL)	52(40–70)	75(53–94)	0.006
IVH, *n* (%)	39 (41.5)	8 (72.7)	0.049
GCS score	7 (5–7)	5 (3–6)	0.003
ICH score	2.5 ± 0.6	3.0 ± 1.0	0.019
**Laboratory values**		
WBC (× 10^9^/L)	12.2 ± 4.6	17.4 ± 9.0	0.002
Neutrophil count (× 10^9^/L)	9.2 ± 4.8	14.2 ± 8.2	0.004
lymphocyte count (× 10^9^/L)	2.3 ± 1.3	1.6 ± 1.2	0.064
NLR (%)	3.8 (1.9–8.5)	8.8 (6.5–15.6)	0.002
D-dimer	3.0 ± 8.0	3.8 ± 2.6	0.742
CRP (mg/L)	14.0 (5.0–54.7)	49.0 (27.9–135.6)	0.012

GCS, Glasgow coma scale; ICH, Intracerebral hemorrhage; IVH, Intraventricular hemorrhage; WBC, White blood cells; NLR, Neutrophil-to-lymphocyte ratio; CRP, C-reactive protein.

To explore the relationship between the risk factors and the outcome prognosis, we performed a correlation analysis. The results showed that the risk factors for 30-day death did not include intraventricular hematoma (*p* = 0.062) but were related to the hematoma volume (*p* = 0.012), GCS score (*p* = 0.005), ICH score (*p* = 0.029), WBC count (*p* = 0.010), neutrophil count (*p* = 0.011), NLR (*p* = 0.001) and CRP level (*p* = 0.018) (Table 2). These risk factors were calibrated for age, gender and GCS score (Table 3).

**Table 2:** Risk factors associated with 30-day death.

**Independent variable**	**OR (95% CI)**	***p*-value**
ICH volume	1.0 (1.0, 1.1)	0.012
IVH	3.8 (0.9, 15.1)	0.062
GCS score	0.6 (0.4, 0.8)	0.005
ICH score	3.0 (1.1, 7.8)	0.029
WBC	1.2 (1.0, 1.3)	0.010
Neutrophil count	1.2 (1.0, 1.3)	0.011
NLR	1.2 (1.1, 1.4)	0.001
CRP	1.0 (1.0, 1.0)	0.018

GCS, Glasgow coma scale; ICH, Intracerebral hemorrhage; IVH, Intraventricular hemorrhage; WBC, White blood cells; NLR, Neutrophil-to-lymphocyte ratio; CRP, C-reactive protein.

**Table 3:** Adjusted risk factors for 30-day mortality in ICH patients.

**Variable**	**Unadjusted**		**Adjusted** ^**a**^	

	**OR (95% CI)**	***p*** **-value**	**OR (95% CI)**	***p*** **-value**
ICH volume	1.0 (1.0, 1.1)	0.012	1.0 (1.0, 1.1)	0.014
GCS score	0.6 (0.4, 0.8)	0.005	0.6 (0.4, 0.8)	0.006
ICH score	3.0 (1.1, 7.8)	0.029	2.9 (1.1, 7.8)	0.032
WBC	1.2 (1.0, 1.3)	0.010	1.2 (1.1, 1.4)	0.007
Neutrophil count	1.2 (1.0, 1.3)	0.011	1.2 (1.0, 1.4)	0.008
NLR	1.2 (1.1, 1.4)	0.001	1.2 (1.1, 1.4)	0.002
CRP	1.0 (1.0, 1.0)	0.018	1.0 (1.0, 1.0)	0.027
**Variable**	**Unadjusted**		**Adjusted** ^b^	
	**OR (95% CI)**	***p*** **-value**	**OR (95% CI)**	***p*** **-value**
ICH volume	1.0 (1.0–1.1)	0.012	1.0 (1.0–1.0)	0.093
ICH score	3.0 (1.1–7.8)	0.029	2.2 (1.0–6.1)	0.152
WBC	1.2 (1.0–1.3)	0.010	1.2 (1.0–1.4)	0.016
Neutrophil count	1.2 (1.0–1.3)	0.011	1.2 (1.0–1.4)	0.015
NLR	1.2 (1.1–1.4)	0.001	1.3 (1.2–1.6)	0.001
CRP	1.0 (1.0–1.0)	0.018	1.0 (1.0–1.0)	0.109

GCS, Glasgow coma scale; ICH, Intracerebral hemorrhage; WBC, White blood cells; NLR, Neutrophil-to-lymphocyte ratio; CRP, C-reactive protein; ^*a*^Adjustment by age and gender; ^*b*^Adjustment by age, gender and GCS score.

To compare the diagnostic efficacy of risk indicators, we analyzed the univariate ROC curve of ICH volume, GCS score, ICH score, WBC count, neutrophil count, NLR and CRP level (Table 4). The AUC and 95% confidence interval of the ICH volume, GCS score, ICH score, WBC count, neutrophil count, NLR, and CRP level were (0.7065, 0.5307–0.8823), (0.7660, 0.6128–0.9191), (0.6248, 0.4312–0.8183), (0.6963, 0.5304–0.8623), (0.6939, 0.5415–0.8463), (0.7901, 0.6754–0.9049), and (0.7311, 0.5869–0.8754), respectively. The results show that the AUC of NLR is the largest.

**Table 4:** Comparisons of risk factors of ICH patients.

	**AUC**	**95%CI**	**Cut-off**	**Specificity**	**Sensitivity**	**Accuracy**	**+LR**	**−LR**
ICH volume	0.7065	0.5307–0.8823	74.0000	0.8298	0.5455	0.8000	3.2045	0.5478
GCS score	0.7660	0.6128–0.9191	5.5000	0.7340	0.7273	0.7333	2.7345	0.3715
ICH score	0.6248	0.4312–0.8183	3.5000	0.9787	0.2727	0.9048	12.8182	0.7431
WBC	0.6963	0.5304–0.8623	13.4050	0.6596	0.7273	0.6667	2.1364	0.4135
Neutrophil count	0.6939	0.5415–0.8463	6.3250	0.3723	1.0000	0.4381	1.5932	0.0000
NLR	0.7901	0.6754–0.9049	5.5250	0.5638	1.0000	0.6095	2.2927	0.0000
CRP	0.7311	0.5869–0.8754	26.0500	0.6064	0.9091	0.6381	2.3096	0.1499
Model 1	0.8385	0.7234–0.9536	−2.6904	0.6277	0.9091	0.6571	2.4416	0.1448
Model 2	0.9304*	0.8450–1.0000	−1.0338	0.9681	0.8182	0.9524	25.6364	0.1878

GCS, Glasgow coma scale; ICH, Intracerebral hemorrhage; WBC, White blood cells; NLR, Neutrophil-to-lymphocyte ratio; CRP, C-reactive protein; AUC, Area under curve; +LR, Positive- likelihood ratio; -LR, Negative- likelihood ratio. Model 1, −4.90143 + 0.01894 ^*^ (ICH volume) −0.22312 ^*^ (WBC) + 0.36191 ^*^ (Neutrophil count) + 0.01277 ^*^ (CRP) + 0.74191 ^*^ (ICH score) −0.38027 ^*^ (GCS score). Model 2, −7.47229 + 0.02976 ^*^ (ICH volume) + 0.54289 ^*^ (WBC) −0.53591 ^*^ (Neutrophil count) + 0.40806 ^*^ (NLR) + 0.00730 ^*^ (CRP) + 0.49988 ^*^ (ICH score) −0.66796 ^*^ (GCS score). ^*^Compared with AUC of Model 1, *p* < 0.05.

We established a mathematical predictive model of ICH to further conduct multivariate logistic regression analyses. Death within 30-days was set as the dependent variable and ICH volume, GCS score, ICH score, WBC count, neutrophil count, and CRP level as the independent variables. The equation of ICH prediction Model 1 was −4.90143 + 0.01894 × (ICH volume) −0.22312 × (WBC) + 0.36191 × (neutral count) + 0.01277 × (CRP) + 0.74191 × (ICH Score) −0.38027 × (GCS score) (Table 5). Then, the independent variable NLR was also introduced on the basis of Model 1, and a logistic regression analysis was carried out to construct multifactor joint prediction Model 2. The regression equation was −7.47229 + 0.02976 × (ICH volume) + 0.54289 × (WBC) −0.53591 × (neutral count) + 0.40806 × NLR + 0.00730 × (CRP) + 0.49988 × (ICH Score) −0.66796 × (GCS score) (Table 5). A ROC curve analysis was applied to compare the diagnostic efficacy of Model 1 and Model 2 (Table 4), which showed that the prognostic efficacy of Model 2 was better than that of Model 1 (AUC: 0.9304 vs. 0.8383, *p* = 0.0270).

**Table 5:** Construction of predictive mathematical models for ICH patients.

	**OR (95% CI)**	***p*-value**
**Model 1**		
ICH volume	1.0191 (0.9892–1.0499)	0.2127
White blood cells	0.8000 (0.4631–1.3821)	0.4238
Neutrophil count	1.4361 (0.8103–2.5451)	0.2151
CRP	1.0128 (0.9979–1.0280)	0.0923
ICH score	2.1000 (0.6034–7.3079)	0.2436
GCS score	0.6837(0.4185–1.1169)	0.1289
**Model 2**		
ICH volume	1.0302 (0.9895–1.0726)	0.1485
White blood cells	1.7210 (0.7937–3.7316)	0.1692
Neutrophil count	0.5851 (0.2505–1.3668)	0.2157
NLR	1.5039 (1.1166–2.0255)	0.0072
CRP	1.0073 (0.9876–1.0274)	0.4683
ICH score	1.6485 (0.3839–7.0793)	0.5014
GCS score	0.5128 (0.2577–1.0202)	0.0570

GCS, Glasgow coma scale; ICH, Intracerebral hemorrhage; NLR, Neutrophil-to-lymphocyte ratio; CRP, C-reactive protein.

## Discussion

Stroke is mainly divided into ICH and ischemic stroke. A study from bigdata observatory platform for stroke of China showed that the ICH group had the highest rate of in-hospital mortality (0.9% for ischemic stroke, 5.1% for ICH). Meanwhile, the 1-year fatality and disability rates of patients with ICH were also higher than those of patients with ischemic stroke (20). Spontaneous intracerebral hemorrhage mostly occurs in the basal ganglia and is related to hypertension, and it presents high disability and mortality rates (21). However, the relevant treatment strategies and clinical prognoses are still controversial (22). Patients with severe basal ganglia hemorrhage have catastrophic outcomes, and the 30-day mortality is over 30% (23). There are two main aspects of brain injury caused by intracerebral hemorrhage: the primary destructive effect of hematoma and secondary injury caused by the space-occupying effect (24). Some studies suggest that the factors related to the prognosis of patients with ICH include the volume of hematoma, the ICH score, and whether the hematoma breaks into the ventricle (14); however, convenient and reliable biological markers for judging the prognosis are lacking.

Inflammatory responses are widely involved in the process of a variety of diseases and can even be used as an independent risk factor for prognosis (25–28). After intravascular recanalization in patients with acute ischemic stroke (AIS), recanalization and reperfusion of previously hypoxic brain regions increase the proinflammatory function of platelets, and the activated thrombus inflammatory reaction may aggravate the ischemia reperfusion injury. Moreover, T cells and platelets will further accelerate the progression of cerebral infarction and expand the infarct area (29). AIS patients who received endovascular therapy and successfully recanalized had higher systemic inflammatory response index (SIRI, SIRI = absolute neutrophil count × absolute monocyte count/ absolute lymphocyte count) at admission, the risk of poor neurological prognosis at 3 months was also increased. SIRI is an independent risk factor for the prognosis of patients with ineffective recanalization (30). Similarly, a retrospective study found that the increase of neutrophil count and NLR before thrombolysis in AIS was independently associated with ICH after thrombolysis and deterioration of prognosis at 3 months (31). Moreover, elevated NLR also indicates an increased risk of symptomatic intracerebral hemorrhage in AIS patients after vascular recanalization therapy (32). In addition, studies on patients with hemorrhagic stroke found that elevated NLR is a good predictor of early neurological deterioration and poor functional status at 3 months (15, 33). Once ICH occurs, the inflammatory reaction is activated, and then inflammatory cells migrate, infiltrate local brain tissue, release cytokines, affect the stability of the blood–brain barrier, aggravate tissue edema, and even lead to hematoma expansion, resulting in a vicious cycle (34, 35). Therefore, evaluating the inflammatory response in patients with ICH will help us to predict the outcomes. The NLR is the ratio of the neutrophil count and lymphocyte count in peripheral blood, which is easy to obtain in the clinic and can be used to dynamically monitor the body's immune ability and systemic inflammatory state. The predictive ability of the NLR for clinical outcome has been verified in a variety of clinical models of brain diseases (9–12). In patients with aneurysmal subarachnoid hemorrhage, NLR is an independent risk factor for poor functional prognosis (10). In addition, NLR is defined as an independent factor for early deterioration of neurological function in patients with AIS after thrombolytic therapy (36). NLR is a promising predictor of clinical outcomes in patients with ischemic and hemorrhagic stroke. Furthermore, increased NLR is associated with a higher risk of ischemic stroke (4). However, the predictive role of the NLR in patients with ICH is still controversial. Several scholars have found that the NLR level was negatively correlated with the short-term prognosis in ICH patients (13–15), and other studies reported that the NLR at admission was not associated with the 30- or 90-day mortality (13, 16). The possible reason for these contradictory results is that the previous studies did not screen and distinguish the bleeding area, hematoma volume and admission status of the selected patients in detail, which seriously affected the prognosis and resulted in biased results. Therefore, if we can improve the homogeneity of enrolled patients, it will improve the accuracy of predictions. In view of this, we established a mathematical model that focuses on patients with severe basal ganglia intracerebral hemorrhage (GCS score ≤ 8 and hematoma volume ≥ 30 cm^3^) and explored the relationship between NLR in peripheral blood routine at admission and 30-day mortality. Briefly, the NLR at admission is positively correlated with the 30-day mortality. To improve our understanding of this dangerous disease and improve the accuracy of disease prediction, we carried out univariate and multivariate logistic regression analyses and established mathematical predictive models. The univariate ROC analysis showed that the ICH volume, GCS, ICH score, WBC, neutrophil count and NLR could be used to predict the 30-day mortality in patients with severe basal ganglia ICH, and NLR was the most reliable. Further construction of the combined predictive model showed that compared with Model 1, predictive Model 2, which was composed of the NLR at admission, was superior to the single NLR and Model 1, and the specificity and accuracy were obviously improved. In conclusion, the NLR is expected to be a potential biomarker for predicting the prognosis of patients with severe basal ganglia hemorrhage.

Some shortcomings were observed in this study. First, this was a retrospective study in which patients were selected from a single center; thus, the screening may be biased. Second, although inflammatory factors may be associated with the enlargement of hematoma and the aggravation of brain edema (34, 37, 38), but we did not detect the expression level of inflammatory factors or their relationship with NLR and prognosis.

Basal ganglia intracerebral hemorrhage is a type of stroke with high mortality and disability rate, and has a trend of youth, which brings heavy burden to society and families. Therefore, how to quickly, accurately and economically identify the risk factors of patients with cerebral hemorrhage is particularly important, which will help to provide reference for follow-up treatment. It is known that the neuroinflammatory reaction triggered by cerebral hemorrhage will lead to a series of reactions and affect the prognosis of patients (39). Neutrophils invade brain tissues at the early stage of lesions, release proinflammatory cytokines and other cytotoxic products, and promote the secondary damage of potential living tissues (40). It can also increase the permeability of blood brain barrier and lead to brain edema after stroke (40). In addition, neutrophils adhere to the blood vessel wall, which can form secondary obstruction in the cerebral microvessels (41). On the contrary, lymphocyte count is considered to have neuroprotective effects and help to improve neural function (42). The peripheral blood NLR is a relatively easy to obtain and widely used clinical marker, which is routinely used to respond to systemic inflammatory reaction. Therefore, the analysis of NLR is helpful to understand the potential pathophysiology of ICH, and can also be used as a factor to predict the prognosis of patients with ICH, providing inspiration for clinical practice and future research of this public health concern.

## Data availability statement

The raw data supporting the conclusions of this article will be made available by the authors, without undue reservation.

## Ethics statement

This study was approved by the Ethics Committee of The Third Affiliated Hospital of Soochow University. All methods were carried out in accordance with relevant guidelines and regulations. Informed consent was obtained from the patient for anonymized information to be published in this study.

## Author contributions

JS was a major contributor to design the study, analyze the data, and draft the manuscript. YL and LW collected and organized the patients' data. WX was patient management. WG participated in the design of the study and coordination of the whole work. All authors read and approved the final manuscript.
